# Artificial Intelligence‐Based Methods: The Path Forward in Achieving Equity in Lung Cancer Screening and Evaluation

**DOI:** 10.1002/cai2.70019

**Published:** 2025-06-20

**Authors:** Stephen J. Kuperberg, David C. Christiani

**Affiliations:** ^1^ Division of Pulmonary and Critical Care Medicine, New York City Health and Hospitals, Woodhull Brooklyn New York USA; ^2^ New York University Grossman School of Medicine New York New York USA; ^3^ Harvard T. H. Chan School of Public Health, Harvard Medical School, Massachusetts General Hospital Boston Massachusetts USA

**Keywords:** artificial intelligence, barriers, disparities, equity, lung cancer, machine learning, natural language processing, socioeconomic

## Abstract

Although lung cancer remains a global threat to public health, evidenced based advances in screening and prevention hold promise for reducing its impact on mortality. An ongoing challenge facing the clinical and research community are the glaring disparities in access to preventive services faced by ethnically and socioeconomically marginalized groups. In this context, novel approaches are needed to improve research methods and thus bolster our ability to improve outcomes. Artificial intelligence (AI) applications such as machine learning and natural language processing hold promise as catalysts in this process, enhancing speed, accuracy and capability. This perspective will highlight the potential of AI methods as essential tool for growth across the lung cancer diagnostic continuum from screening to diagnosis.

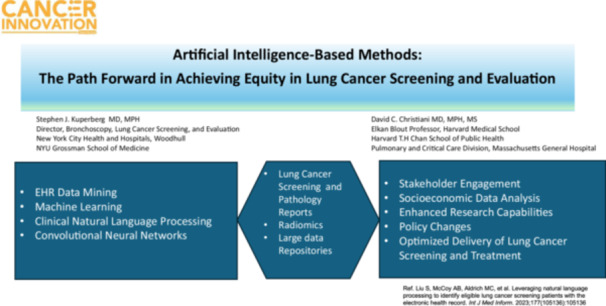

**Abbreviations**
AI: artificial intelligence
LCS: lung cancer screening
NLP: natural language processing

## 1 The Evolution of Lung Cancer Screening

Despite the paradigm shift in treatment modalities driven by immunology, mortality from lung cancer remains the highest of all cancers [1], rendering it an unrelenting and formidable public health threat. Fortunately, coordinated global efforts have been made, both in the United States and internationally to reduce lung cancer mortality via primary and secondary screening measures, that is, smoking cessation and population screening [2].

In the United States alone, national initiatives have emerged to propel the lung cancer screening (LCS) agenda forward such as the U.S. Department of Health and Human Services Healthy People 2030 vision and the Cancer Moonshot Initiative [3]. LCS has emerged as a new tidal front in cancer control following the publication of robust data from large‐scale trials demonstrating a mortality benefit [4, 5]. This agenda was advanced further in 2022, when the Center for Medicare and Medicaid Services expanded eligibility for adults being screened—lowering the minimum age to 50, while reducing the required number of pack years of tobacco smoking from 30 to 20 in active smokers or those who have quit in the past 15 years.

These interventions have already yielded a significant public health impact, reducing mortality globally while improving our ability to capture lung malignancies at an earlier stage leading to curative surgery.

## 2 Roadblocks to LCS Equity

Still, challenges in implementation loom. Expansion of LCS exposes the need for new funding and policy change to accommodate the massive influx of eligible individuals, which is recently estimated to be greater than 5 million individuals [6]. Moreover, a persistent gap exists in the capacity for current LCS and nodule surveillance programs to reach populations equally and equitably [7, 8, 9]. Emerging data have supported the influential role of geographic area of residency as a key access factor, often conceptualized as “neighborhood disadvantage” [10]. In this construct, high‐risk patients, particularly from Black and Latinx neighborhoods, are more likely to face formidable challenges in equitable diagnosis and screening participation, such as underscreening, the receipt of guideline discordant care, and increased time to diagnosis [8].

For example, despite the known survival benefit associated with LCS, only 10%–20% of eligible Hispanic and Latinx individuals undergo screening [11], and are less likely to be adherent (with reports as low as 9% reported in candidates enrolled in decentralized LCS programs), risking late stage diagnosis and mortality. The underlying reasons for poor uptake within this population are complex, including structural racism and social and cultural factors [12], also underscoring the vital need for further study of improved methods for optimal data collection. There is strong evidence that vulnerable groups with non‐small cell lung cancer are less likely to receive safer, novel management options such as immunotherapy [13], and patients within these groups are less likely to undergo resection. The result of these converging factors is a significant survival disadvantage in lung cancer, which is associated with poverty and racial disparity.

## 3 AI can Improve Research and Data Collection Methods in LCS

It is not surprising, therefore, that in the wake of numerous studies demonstrating stark ethnodemographic disparities in LCS outcomes [7], major society guidelines have emphasized the importance of efforts to mitigate inequity. The American Thoracic Society has addressed equity concerns in both the screening and nodule surveillance domains, wherein barriers to implementation of LCS programs can be categorized based on patient, provider, and institutional levels [14, 15]. Indeed, the socioeconomic spectrum of income, race, and ethnicity is linked to reduced uptake and utilization of LCS recommendations [8].

For the aforementioned reasons, new technological avenues to improve data collection will be essential for the scientific community to continue developing to reach diverse ethnic and racial patient populations [7, 15, 16]. The recent emergence of artificial intelligence (AI) methods, such as natural language processing (NLP), large language models, and machine learning, is increasingly implemented in the sphere of lung cancer detection and surveillance. These tools have the advantage of being applied to both image analysis and clinical testing. Large patient and population EHR datasets are fertile ground for mining clinical, sociodemographic information, wherein rapidly assessing baseline characteristic associations and outcomes, and lead to public health change [17, 18].

A key example of an application that can catalyze LCS and lung cancer risk prediction is the use of “clinical” natural language processing, or “cNLP” of electronic health record data [19]. Parameters related to social determinants of health [20]—in addition to clinical risk factors—can be extracted and analyzed with respect to the patient's location, income, and insurance status [21] and the information mobilized to determine associations and thus inform policy and potentially reduce socioeconomic barriers to lung cancer survival. Notably, a recent feasibility study published by Liu et al. validated a novel NLP‐based approach that successfully extracted both LCS eligibility and sociodemographic information from electronic health records in a large academic medical center [9]. Moreover, AI tools can be interfaced with image analysis, that is, “radiomics” [22] the latter of which employs morphologic feature extraction, and can be integrated into predictive models for rapid, accurate analysis of the computed tomography scans generated by lung screening and lung nodule programs [16] and merged with demographic data. This area of overlap represents a gap in the current literature, revealing a clear need for future studies.

## 4 Stakeholder Involvement Is Essential to Reduce Disparities

Ultimately, diverse stakeholders must be engaged to successfully bridge the divide between healthcare disparities and implementation [23, 24] and realize the potential for lung screening to reduce mortality. Policy makers, hospital administrators, and institutional leadership must employ both top‐down and horizontal collaborative approaches. An example of this is seen in the National Lung Cancer Roundtable held by the American Cancer Society [25] where key stakeholders in LCS convene to refine strategic priorities and bolster implementation of LCS including enhancement of outreach, engage providers, and ultimately expand LCS programs.

It is imperative for the research and clinical communities to forge high‐value collaborations with information technology specialists who possess the technological savvy to integrate novel software into EHR, and collaborate with multiple specialties and primary care clinicians who can then interface with patients on the front line. Finally, an international approach whereby AI‐driven registry data can be shared in an “open library approach” can enhance the understanding of risk levels and potentially highlight differences in screening outcomes across populations [26].

## 5 A Call to Action for Utilizing AI to Reduce LCS Disparities

In summary, AI technologies will transform reporting, collecting, and processing population data, whether in public datasets and repositories or within institutions, paving the way for discovery and methodology development in lung cancer detection. Given the vast body of evidence underscoring social disparities and their influence on lung cancer outcomes, it is imperative to move forward to evaluating and implementing AI research methods that have the capacity to improve our ability to detect malignant lesions at an early stage and reduce mortality. Thus, appropriately harnessing these tools in the clinic and in well‐designed population studies is an important next step. Doing so will allow us to seamlessly move forward in our aim of overcoming healthcare inequity in lung cancer evaluation on multiple levels.

## Author Contributions

**Stephen J. Kuperberg:** conceptualization (equal), formal analysis (equal), writing – original draft (equal), writing – review and editing (equal). **David C. Christiani:** conceptualization (equal), data curation (equal), formal analysis (equal), writing – original draft (equal), writing – review and editing (equal).

## Ethics Statement

The authors have nothing to report.

## Consent

The authors have nothing to report.

## Conflicts of Interest

Dr. Kuperberg has received consulting fees from Merck. Dr. Christiani has no relevant conflicts of interest to report pertaining to the content of this manuscript.

## Data Availability

The authors have nothing to report.
